# Prediction value of the genetic risk of type 2 diabetes on the amnestic mild cognitive impairment conversion to Alzheimer’s disease

**DOI:** 10.3389/fnagi.2022.964463

**Published:** 2022-09-15

**Authors:** Jiayang Yang, Zirui Wang, Yumeng Fu, Jiayuan Xu, Yang Zhang, Wen Qin, Quan Zhang

**Affiliations:** Department of Medical Imaging and Tianjin Key Laboratory of Functional Imaging, Tianjin Medical University General Hospital, Tianjin, China

**Keywords:** cortical volume, gray matter volume, magnetic resonance imaging, mediation analysis, polygenic risk score

## Abstract

Amnestic mild cognitive impairment (aMCI) and Type 2 diabetes mellitus (T2DM) are both important risk factors for Alzheimer’s disease (AD). We aimed to investigate whether a T2DM-specific polygenic risk score (PRS_*sT2DM*_) can predict the conversion of aMCI to AD and further explore the underlying neurological mechanism. All aMCI patients were from the Alzheimer’s disease Neuroimaging Initiative (ADNI) database and were divided into conversion (aMCI-C, *n* = 164) and stable (aMCI-S, *n* = 222) groups. PRS_*sT2DM*_ was calculated by PRSice-2 software to explore the predictive efficacy of the aMCI conversion to AD. We found that PRS_*sT2DM*_ could independently predict the aMCI conversion to AD after removing the common variants of these two diseases. PRS_*sT2DM*_ was significantly negatively correlated with gray matter volume (GMV) of the right superior frontal gyrus in the aMCI-C group. In all aMCI patients, PRS_*sT2DM*_ was significantly negatively correlated with the cortical volume of the right superior occipital gyrus. The cortical volume of the right superior occipital gyrus could significantly mediate the association between PRS_*sT2DM*_ and aMCI conversion. Gene-based analysis showed that T2DM-specific genes are highly expressed in cortical neurons and involved in ion and protein binding, neural development and generation, cell junction and projection, and PI3K-Akt and MAPK signaling pathway, which might increase the aMCI conversion by affecting the Tau phosphorylation and amyloid-beta (Aβ) accumulation. Therefore, the PRS_*sT2DM*_ could be used as a measure to predict the conversion of aMCI to AD.

## Introduction

Alzheimer’s disease (AD), a neurodegenerative disease, is the main cause of dementia. The etiology of AD is unclear; some researches supports that it might be related to multiple factors such as genetics, living habits, age, and education (Norton et al., 2014; Adams et al., 2015; Cuyvers and Sleegers, 2016). Today, there is no effective cure for AD, which burdens society and families. Therefore, early prevention has become an important measure to reduce AD’s prevalence. Amnestic mild cognitive impairment (aMCI) is a cognitive deficit with memory impairment as the main manifestation (Winblad et al., 2004). Patients with aMCI have a higher risk of converting to AD, and approximately 12% progress to AD yearly (Palmer et al., 2008). Therefore, finding the risk factors that can predict aMCI conversion to AD is important for early AD prevention (Xu et al., 2018).

Previous studies have shown that education level (Tokuchi et al., 2014), clinical history (Agostini et al., 2016), neuropsychology (Mazzeo et al., 2016; Jang et al., 2017), cognitive behavior level (Julayanont et al., 2014), genetics (Adams et al., 2015; Xu et al., 2018), and neuroimaging (Yuan et al., 2009) can be used to predict aMCI conversion to AD. As a risk factor for AD, Type 2 diabetes mellitus (T2DM) (Ma et al., 2015) and blood glucose levels (Albai et al., 2019) also had predictive values for the aMCI conversion to AD. Individuals with both T2DM and APOE 4 allele had a risk ratio (RR) of 5.5 for AD compared to those with neither (Peila et al., 2002). T2DM and AD share some similar pathological findings, such as decreased insulin expression level, insulin-like growth factor 1 (IGF-1), insulin receptor substrate, and increased amyloid precursor protein (APP) expression level (de la Monte and Wands, 2005). The deficiency of insulin-PI3K-AKT signaling was more severe in individuals with T2DM and AD than in those with either alone. The level of PI3K-AKT signaling was negatively correlated with tau phosphorylation (Liu et al., 2011). Deposition of Aβ in brain and islet cells exhibits similar pathogenicity in T2DM and AD (Beeler et al., 2009). Autopsy studies in T2DM patients have shown that amyloid plaques and neurofibrillary tangles are also present in the hippocampus of T2DM patients (Peila et al., 2002). All these studies demonstrated a link between T2DM and AD (Hossain et al., 2020), and T2DM is the risk factor for AD. Genetic factors play an important role in the pathogenesis of T2DM (Sladek et al., 2007) and AD (Lambert et al., 2013; Cuyvers and Sleegers, 2016). Genome-wide association studies (GWAS) have found that the occurrence of T2DM and AD is associated with multiple single nucleotide polymorphisms (SNPs), and they have many shared genetic variation sites (Wang et al., 2017). A longitudinal study showed that the T2DM risk SNP (rs391300) could predict aMCI’s conversion to AD (Girard et al., 2018). However, it is still not clear whether the cumulative genetic risks of T2DM can improve the ability to predict aMCI’s conversion to AD. Thus, this study aimed to investigate if the cumulative T2DM-specific genetic risks could predict the conversion from aMCI to AD. If so, we further explored the possible neurobiological mechanisms underlying the predictive effect.

In this study, a polygenic risk score (PRS) was used to assess the accumulated genetic risks for T2DM and AD. The PRS is one of the best indicators for evaluating the polygenic risk of disease and has been used to predict disease conversions, such as using the PRS of AD (PRS_*AD*_) (Adams et al., 2015) and major depression (PRS_*MDD*_) (Xu et al., 2018) to predict the aMCI conversion to AD. It is well-known that brain atrophy is one of the main features of AD (Fox and Schott, 2004). Multiple studies have reported that relatively reduced brain volumes in both aMCI and AD patients, including the hippocampus, parahippocampal gyrus, cingulate, and other brain regions (Jack et al., 1999, 2008; Fox and Schott, 2004; Karas et al., 2004), and the atrophy of brain structure can be used as the biomarker to predict the conversion of aMCI to AD (Visser et al., 2002; Stoub et al., 2005; Yuan et al., 2009; Risacher and Saykin, 2013). Xu et al. (2018) further found that the left hippocampal volume mediates the predictive effect of the PRS_*MDD*_ on the conversion of aMCI to AD. However, no studies have reported whether brain structures could mediate the predictive effect of the T2DM genetic variants on the conversion of aMCI to AD. Mediation analysis was widely used to explore the underlying mechanism of a known relationship: one variable influences another variable through a mediator variable (VanderWeele, 2016). Firstly, we used the genetic variants specific to T2DM to calculate PRS_*sT2DM*_ and investigate if the PRS_*sT2DM*_ could predict the conversion from aMCI to AD after excluding the common genetic variants with AD. Secondly, mediation analysis was used to assess whether the PRS_*sT2DM*_ effect on the prediction was mediated by the brain structures, including voxel-based morphometry (VBM) and surface-based morphology (SBM). Finally, genetic variants for calculating the PRS_*sT2DM*_ and genetic variants in GWAS of T2DM were fine-mapped into genes. Then enrichment analysis of those overlapped genes was conducted to identify the potential functions of these genes. In addition, cell type-specific expression analysis was performed to explore the specific cell types these genes are significantly expressed.

## Materials and methods

### Base dataset and target dataset

During the PRS calculation, a base dataset was needed to calculate the genetic effect size associated with the disease status at a predefined threshold. In this study, GWAS data of the Diabetes Genetics Replication and Meta-analysis (DIAGRM) (Mahajan et al., 2018) and International Genomics of Alzheimer’s Project (IGAP) (Lambert et al., 2013) were used as the base dataset to calculate PRS_*T2DM*_ and PRS_*AD*_ in a target dataset, respectively. The target dataset provided by all stages of Alzheimer’s Disease Neuroimaging Initiative (ADNI1/GO/2/3).^1^ There are 975 patients in total with the baseline diagnosis of aMCI. Diagnosis of aMCI was made according to the criteria by Petersen (Petersen et al., 1999). Among them, 522 patients had no whole-genome sequencing information, five patients had no follow-up information, one patient’s follow-up was too short (≤3 months), 52 patients converted into normal controls during the follow-up, eight patients converted into AD then back to aMCI during the follow-up, and one patient’s structural MRI image could not be downloaded. After excluding the above subjects, 386 aMCI patients’ data were retained in the subsequent statistical analyses. According to the follow-up outcome in March 2019 (the follow-up time range is 6–156 months, an average of 50.69 months), 386 aMCI patients were divided into conversion (aMCI-C, *n* = 164) and stable (aMCI-S, *n* = 222) groups. The final clinical diagnosis will be based on the last follow-up results if the subject is missing during the follow-up.

### Genotyping and quality control

The whole-genome sequencing was performed on Illumina HiSeq2000 platform. The genome-wide SNPs were genotyped using the Illumina Omni 2.5M Bead Chip for the ADNI subjects.

Quality control was performed at both the individual level and SNP level. Subjects with a missing genotyping rate of >0.05, sex inconsistency, possible relative relationships, and European population outliers identified by multidimensional scaling (MDS) were excluded. The first four components of MDS analysis were used as covariates in subsequent analysis. SNPs with a missing call rate of >0.05, minor allele frequency <0.01, a significant deviation from Hardy-Weinberg equilibrium (*P* < 5 × 10^–6^), and ambiguous strands were excluded. Finally, 9,845,494 SNPs from 386 subjects were included in the subsequent analyses.

### Polygenic risk score calculation

In the base dataset, the associations between the SNPs and disease status were calculated at predefined *P* threshold (*P*_*T*_) values ranging from 5 × 10^–5^ to 0.5 with an increment of 5 × 10^–5^. Under each *P*_*T*_ value, we removed the effects of SNPs in linkage disequilibrium (LD) in each clumped region (excluding SNPs with r^2^ > 0.1, within a 250-kb window) and selected the index SNPs (iSNPs) with the most significant *P*-value from each clumped association region. Thus, the information of the risk alleles and effect sizes of the iSNPs were obtained for each *P*_*T*_ value. In the target dataset, the PRSice-2 (v2.2.6) software (Choi and O’Reilly, 2019) was used to calculate the PRS according to Equation 1.


(1) $$\text{PRS}=\sum_{\text{i}}\frac{{\text{S}}_{\text{i}}\times {\text{G}}_{\text{i}}}{\text{M}}$$

S_*i*_ is the number of risk alleles of iSNPs, G_*i*_ is the effect sizes (natural logarithm of the odds ratio) of the iSNPs, and M is the number of iSNPs.

In this study, the logistic regression method was used to assess the predictive effects of the PRSs on the conversion from aMCI to AD after controlling for sex, age, educational years at baseline, the number of APOEε4, and the first four MDS components for population stratification. Nagelkerke’s R^2^ was used to estimate the percentage of the variance predicted by the PRS in the regression model. Thus, we could obtain the best *P*_*T*_ values for calculating PRS with the best predictive abilities in the target dataset. We calculated both PRS_*T2DM*_ and PRS_*AD*_ to explore the predictive effects of the two groups of risk genes on the conversion from aMCI to AD.

In this study, we aimed to evaluate the predictive effect of the PRS specific to T2DM (PRS_*sT2DM*_) on the conversion from aMCI to AD. Therefore, we removed the common genetic variants of T2DM and AD and reconstructed PRS_*sT2DM*_ and PRS_*sAD*_. Finally, the four groups of PRS values were z-transformed before being used in the statistical analyses.

### Magnetic resonance imaging data acquisition and preprocessing

All imaging data are publicly available and were downloaded from the ADNI website (see text footnote 1). Following the ADNI acquisition protocol, three-dimension T1 weighted imaging (3D-T1WI) was scanned by 1.5T (ADNI1) or 3.0T (ADNIGO/2) MR scanner with a magnetization prepared rapid gradient echo (MPRAGE) sequence at 54 sites. Image corrections involved calibration, geometry distortion, and reduction of the intensity of non-uniformity applied to each image by the ADNI. We downloaded the corrected 3D-T1WI images of 386 aMCI patients for subsequent analysis.

Statistical Parametric Mapping software package (SPM12^2^) and the voxel-based morphometry (VBM) toolbox CAT12.6-rc1^3^ were used to preprocess the 3D-T1WI images. The preprocessing processes included bias-field-corrected, tissue-classified, DARTEL-based spatial normalization, segmentation, modulation, and smooth with a Gaussian kernel with a full width at half maximum of 8 mm.

FreeSurfer (version 5.3.0^4^) was used to reconstruct the cerebral cortex using the SBM method. In brief, 3D-T1WI images of all subjects were registered to the MNI305 template with an affine way, and then the skulls were stripped. White matter and pial surfaces were constructed with a triangle area called a vertex unit. We visually inspected all images and segmentation quality and manually edited them as necessary.^5^ Vertex-wise cortical thickness was obtained by calculating the shortest distance between the pial and white surface. Vertex-wise surface area was calculated by assigning one-third of each triangle’s area to each of its vertices. Vertex-wise cortical volume was calculated by multiplying the surface area by cortical thickness (Fischl and Dale, 2000; Han et al., 2006). The cortical thickness, surface area, and cortical volume were resampled at 1 mm resolution and smoothed with a Gaussian kernel with a full width at half maximum of 10 mm.

### Statistical analysis

#### Demographic analysis

The Statistical Package for the Social Sciences (SPSS, Armonk, NY, United States: IBM Corp) version 22.0 software package was used for demographic analysis. The chi-square test was used to compare the gender, APOEε4 carrier state, and diabetes condition between the aMCI-C and aMCI-S groups. The independent-sample *t*-test was used to compare the differences in age, educational years, fasting blood glucose levels, and PRS values between the two groups. Considering that the Aβ and the tau protein are the main AD markers, we further compared the difference in the Aβ and tau level of the cerebrospinal fluid (CSF) biomarkers between the aMCI-S and aMCI-C groups. The statistical significance threshold was set at *P* < 0.05.

#### Polygenic risk score analysis

The logistic regression method was used to test the predicting effect of PRS on the conversion from aMCI to AD after controlling for sex, age, educational years at baseline, the number of APOEε4, and the first four MDS components for population stratification. The receiver operating characteristic (ROC) curves were built with the four groups of PRS as independent variables, and the areas under the ROC curves (AUC), sensitivities, and specificities were calculated. Considering the possible impact of the Aβ and tau level on the results, we further performed the logistic regression in 299 patients with the Aβ and tau data after extra controlling the Aβ and tau level.

To clarify the associations between the PRS levels and conversion rates and conversion risks, the 386 aMCI patients were trisected into three groups according to PRS_*sT2DM*_ and PRS_*sAD*_ values, respectively. The bottom third (129 patients) was defined as the low-risk group, the middle third (129 patients) as the middle-risk group, and the upper third (128 patients) as the high-risk group. The chi-square test was used to compare the difference in conversion rate from aMCI to AD among the three groups. Besides, survival analysis was conducted using a Cox proportional hazards model with the PRS_*sT2DM*_ and PRS_*sAD*_ groups (low/middle/high) as independent variables, age, education years, gender, and APOEε4 carrier status as covariates. Finally, hazard ratios (HRs) were used to estimate the predictive effect.

#### Imaging analysis

Voxel-based morphometry analysis was performed using SPM12; Surface-based morphometry (SBM) was analyzed using the QDEC package of FreeSurfer software version 5.3. A multiple regression analysis was conducted to identify the brain regions whose GMV, cortical thickness, surface area, or cortical volume were significantly correlated with the PRS_*sT2DM*_ while controlling for the age, gender, education, APOEε4 carrier status, the first four components of MDS, MRI field strength, and data collection sites. Monte Carlo simulation was used to correct multiple comparisons with the voxel- and vertex-level thresholds of *P* < 0.001, simulation times of 5,000, and cluster-level *P* < 0.05. The brain areas significantly correlated with the PRS_*sT2DM*_ were defined as the regions of interest (ROI). The average values of the structural indices within the ROI were extracted for subsequent ROI-based correlation and mediation analysis.

#### Mediation analysis

The PROCESS (Hayes, 2013) toolkit plugged in SPSS 22.0 was used for mediation analysis. We defined the PRS_*sT2DM*_ as the independent variable, the imaging indices within each ROI as the mediation variable, and the aMCI conversion status (aMCI-S vs. aMCI-C) as the binary dependent variable. We tested the mediation effect with bias-corrected bootstrap, bootstrap samples were 5,000, and CI was 95%. When the 95% CI does not contain zero, it is considered a significant mediating effect. The percentage of interpretable variance by mediation effect was calculated as indirect effect size divided by total effect size.

#### Fine-mapping type 2 diabetes mellitus-specific genetic variants into genes

The T2DM-specific genetic variants were fine-mapped into genes using the software MAGMA (de Leeuw et al., 2015) to evaluate the biological function of these genetic variants. We specified a window size in five kilobases upstream and downstream according to the UCSC Genome Browser GRch37/hg19 release.^6^ Meanwhile, the genetic variants of the GWAS data of the Diabetes Genetics Replication and Meta-analysis (DIAGRM) were fine-mapped into genes (Mahajan et al., 2018) with a threshold of *P* < 0.05 using MAGMA. The overlapped genes of the above two groups of genes were used in the following analysis.

#### Gene enrichment analyses of gene ontology and kyoto encyclopedia of genes and genomes

For the identified genes, enrichment analyses for gene ontology (GO) and Kyoto Encyclopedia of Genes and Genomes (KEGG) were performed using an open online tool, g:Profiler (Raudvere et al., 2019).^7^ All enriched GO and KEGG terms were Bonferroni corrected using a threshold of *P* < 0.05 for statistical significance.

#### Cell type enrichment analysis

The online cell type-specific expression analysis (CSEA) tool (Dougherty et al., 2010)^8^ was used to identify the cell types in which the fine-mapped genes were specifically expressed. Fisher’s exact test was used in the tool, and false discovery rate (FDR) was used for correcting multiple comparisons (Benjamini and Hochberg method, *P* < 0.05). The specificity index probability (pSI) values were set at 0.05, 0.01, 0.001, and 0.0001, respectively.

## Results

### Demographic analysis

The demographic and PRS results are shown in Table 1. There was no significant difference in gender, education, blood glucose level, and diabetes condition between the aMCI-C and aMCI-S groups (*P* > 0.05). The age of the aMCI-C group was slightly older than that of the aMCI-S group (*P* < 0.05). The proportion of APOEε4 carrier and the four groups of PRS were higher in the aMCI-C group than in the aMCI-S group (*P* < 0.001). There were significant differences in the Aβ and tau levels in the CSF between aMCI-S and aMCI-C groups (Supplementary Table 1).

**TABLE 1:** Intergroup comparisons of the demographic information and polygenic risk score (PRS) values.

Variables	aMCI-S (*n* = 222)	aMCI-C (*n* = 164)	Statistics	*P*
Males, *n*	136	100	0.003^a^	0.955
Age at baseline, years	72.48 (7.37)	73.96 (6.78)	−2.018^b^	**0.044**
Educational years	15.89 (2.95)	16.02 (2.91)	−0.418^b^	0.676
Blood glucose, mmol/L	5.68 (1.04)	5.59 (0.93)	0.832^b^	0.406
Diabetes condition	19	16	0.164^a^	0.685
APOE 4 carriers, *n*	80	106	30.897^a^	**<0.001**
PRS_*T2DM*_	−0.17 (0.99)	0.22 (0.98)	−3.864^b^	**<0.001**
PRS_*sT2DM*_	−0.17 (0.99)	0.23 (0.97)	−3.963^b^	**<0.001**
PRS_*AD*_	−0.20 (0.95)	0.27 (1.00)	−4.714^b^	**<0.001**
PRS_*sAD*_	−0.21 (0.95)	0.28 (1.00)	−4.866^b^	**<0.001**

Data are shown as mean (SD) or number. The PRS are z-transformed. *P*-values in bold indicate there are significant differences between groups.

^a^χ^2^ value.

^b^T value.

### PRS_*T2DM*_ could predict the amnestic mild cognitive impairment conversion to Alzheimer’s disease

**FIGURE 1:**
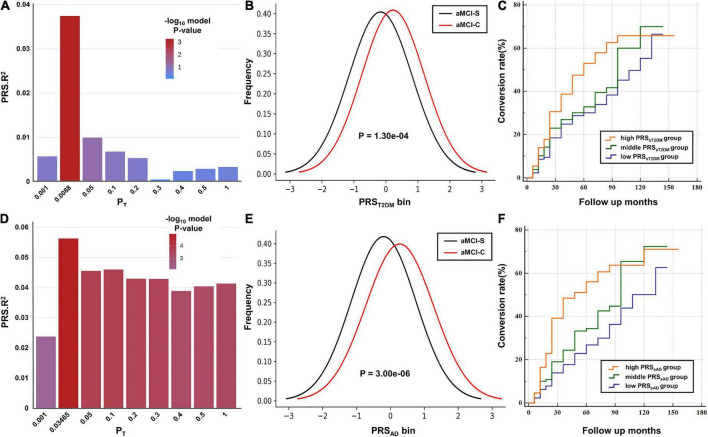
The predictive effects of polygenic risk scores (PRSs) on the amnestic mild cognitive impairment (aMCI) to Alzheimer’s disease (AD). **(A,D)** The bar plots show the predictive effects of the PRS constructed by the best-fit P_*T*_ and other eight broad P_*T*_ values (0.001, 0.05, 0.1, 0.2, 0.3, 0.4, 0.5, and 1) on the conversion of aMCI. The y-axis shows PRS Nagelkerke’s pseudo R^2^ of the predicted model. The color bar shows the logarithm of *P*-value. **(B,E)** The y-axis shows the frequency of each PRS bin (x-axis). **(C,F)** Cox proportional hazard model shows the associations between the PRS and the conversion rate (y-axis) at different time points (x-axis). The orange, green and purple lines show the low, middle and high PRS groups, respectively.

When the T2DM-GWAS data was taken as the base dataset, the PRS_*T2DM*_ calculated at P_*T*_ = 0.0088 exhibited the best predictive effect on the aMCI conversion to AD (*P* = 6.86 × 10^–4^) (Figure 1A, the detailed information at different *P*_*T*_ values are listed in the Supplementary Table 2) and explained 3.74% of variance based on 22,763 index SNPs (Table 2). In addition, the PRS_*T2DM*_ in the aMCI-C group was significantly higher than in the aMCI-S group (Table 1 and Figure 1B).

**TABLE 2:** The predictive effects of polygenic risk score (PRS) on the conversion of amnestic mild cognitive impairment (aMCI) to Alzheimer’s disease (AD).

PRS	P_*T*_	Full.R^2^	PRS.R^2^	PRS.P	iSNPs	Specificity	Senstivitity	AUC	ROC.P
PRS_*T2DM*_	0.0088	17.33%	3.74%	**<0.001**	22763	0.703	0.518	0.613	**<0.001**
PRS_*AD*_	0.03645	19.75%	6.15%	**<0.001**	29321	0.486	0.744	0.637	**<0.001**
PRS_*sT2DM*_	NA	17.53%	3.93%	**<0.001**	22526	0.698	0.518	0.616	**<0.001**
PRS_*sAD*_	NA	20.04%	6.44%	**<0.001**	29084	0.658	0.579	0.640	**<0.001**

P_T_, P-values threshold of genome-wide association studies; Full.R^2^, full Nagelkerke’s pseudo R^2^ of logistic regression; PRS.R^2^, PRS Nagelkerke’s pseudo R^2^ of logistic regression; PRS.P, P-value of PRS prediction model; iSNPs, numbers of single-nucleotide polymorphisms that constitute PRS; AUC, area under the ROC curve; ROC.P, *P*-value of ROC curve. *P*-values in bold indicate there are significant.

The PRS_*AD*_ calculated at P_*T*_ = 0.03645 showed the best predictive effect on the aMCI conversion to AD (*P* = 1.45 × 10^–5^) (Figure 1D) and explained 6.15% of variance based on 29,321 index SNPs (Table 2). The PRS_*AD*_ in the aMCI-C group was significantly higher than in the aMCI-S group (Table 1 and Figure 1E).

### PRS_*sT2DM*_ could independently predict the amnestic mild cognitive impairment conversion to Alzheimer’s disease

After the common genetic variants (*n* = 237) of the two diseases were removed, the PRS_*sT2DM*_ still exhibited a significant predictive effect on the aMCI conversion (*P* = 5.06 × 10^–4^) and explained 3.93% of the variance, which indicated T2DM-specific genetic variants could independently predict the conversion from aMCI to AD. After extra controlling the Aβ and tau level in the CSF, logistic regression analysis showed the PRS_*sT2DM*_ still exhibited a significant predictive effect on the aMCI conversion to AD (β = 0.461, *P* = 0.001). The PRS_*sAD*_ could also independently predict the aMCI conversion (*P* = 9.26 × 10^–6^) and explain 6.44% of the variance.

**FIGURE 2:**
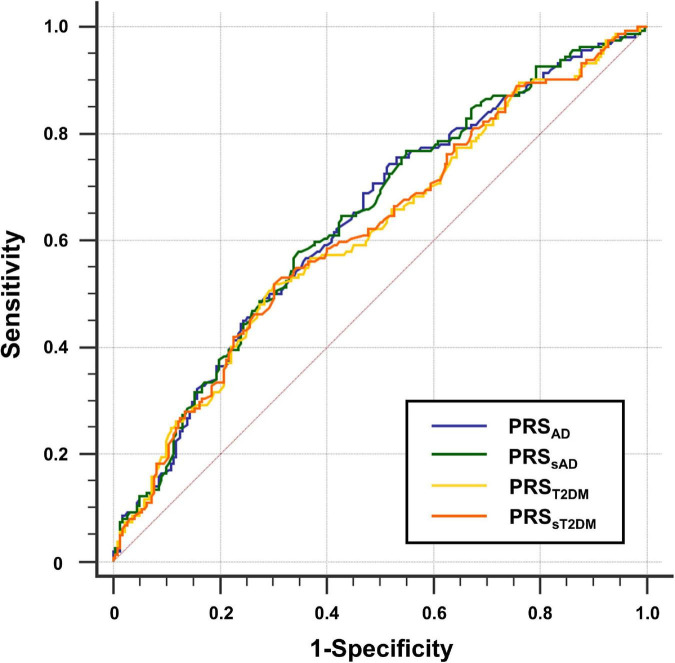
Receiver operating characteristic (ROC) curve for four groups of polygenic risk score (PRS). The y-axis shows sensitivity, the y-axis shows 1-specificity.

The ROC curves of the four PRSs predictive models are shown in Figure 2, and the AUC, sensitivities, and specificities are listed in Table 2.

### Differences of amnestic mild cognitive impairment conversion rates between polygenic risk score subgroups

**FIGURE 3:**
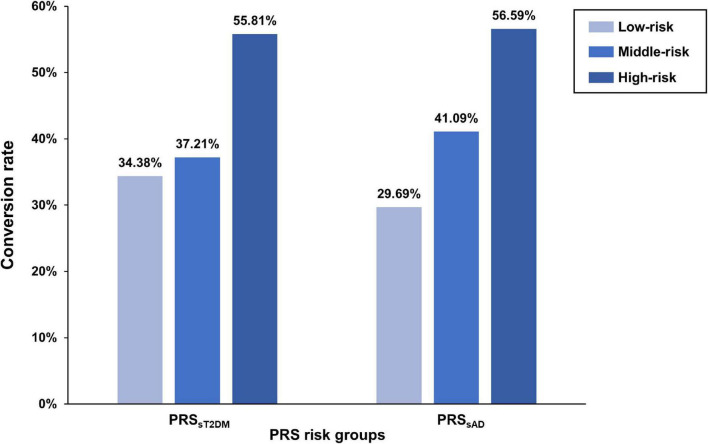
The difference of amnestic mild cognitive impairment (aMCI) conversion rate in polygenic risk score (PRS) risk groups. The bar plots show the conversion rates (y-axis) of each PRS risk group (x-axis).

According to the PRS_*sT2DM*_ and PRS_*sAD*_, 386 aMCI patients were divided into low-, middle-, and high-risk subgroups, respectively. The three PRS_*sT2DM*_ subgroups exhibited a significant difference in the aMCI conversion rate (χ^2^ = 14.30, *P* = 7.87 × 10^–4^). A significant difference in aMCI conversion rate in PRS_*sAD*_ subgroups was also found (χ^2^ = 19.18, *P* = 6.83 × 10^–5^) (Figure 3). Cox survival analysis showed that the risks of aMCI conversion to AD in the middle PRS_*sT2DM*_ group (HRs, 0.67; 95% CI, 0.46–0.97; *P* = 0.034) and the low PRS_*sT2DM*_ group (HRs, 0.60; 95%CI, 0.41–0.88; *P* = 0.009) were lower than in the high PRS_*sT2DM*_ group (Figure 1C). Additionally, aMCI patients in the high PRS_*sT2DM*_ group converted to AD about 5 months on average earlier than the middle PRS_*sT2DM*_ group and a mean of 7 months earlier than the low PRS_*sT2DM*_ group (average 33.67, 38.25, and 40.10 months, respectively). Accordingly, the risk of aMCI conversion to AD in the middle PRS_*sAD*_ group (HRs = 0.66, 95%CI = 0.47–0.95, *P* = 0.024) and the low PRS_*sAD*_ group (HRs = 0.50, 95%CI = 0.34–0.75, *P* = 0.001) was lower than in the high PRS_*sAD*_ group (Figure 1F). aMCI Patients in the high PRS_*sAD*_ group converted to AD about 13 months on average earlier than the middle PRS_*sAD*_ group and a mean of 15 months earlier than the low PRS_*sAD*_ group (average 29.10, 42.00, and 44.05 months, respectively).

### The relationship between the PRS_*sT2DM*_ and gray matter structure

**FIGURE 4:**
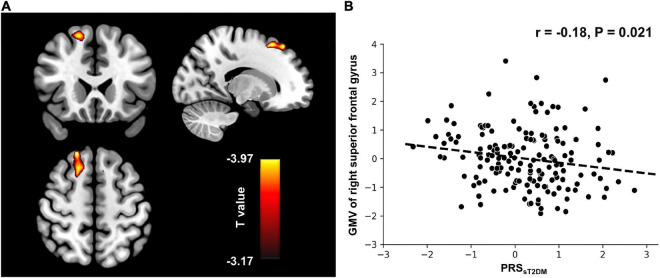
Voxel-based morphometry (VBM) analysis. **(A)** The GMV of the right superior frontal gyrus shows negative correlation with the PRS_*sT2DM*_ in the aMCI-C groups (cluster level, *p* < 0.05). The color bar represents the T value. **(B)** Regions of interest (ROI)-based correlation analysis between the PRS_*sT2DM*_ and the GMV of the right superior frontal gyrus. GMV, gray matter volume.

**FIGURE 5:**
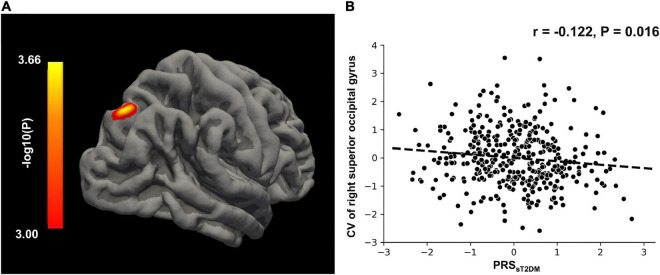
Surface-based morphometry (SBM) analysis. **(A)** The CV of right superior occipital gyrus shows negative correlation with the PRS_*sT2DM*_ in the all aMCI patients (cluster level, *p* < 0.05). The color bar represents the logarithm of *P*-value. **(B)** Regions of interest (ROI)-based correlation analysis between the PRS_*sT2DM*_ and the CV of right superior occipital gyrus. CV, cortical volume.

In the aMCI-C group, the PRS_*sT2DM*_ was negatively correlated with the GMV of the right superior frontal gyrus (MNI coordinates: x = 18, y = 36, z = 55.5; *t* = −3.97; 458 voxels, cluster-level *P* < 0.05) (Figure 4A). The ROI-based correlation analysis is shown in Figure 4B. A significant negative correlation was found between the PRS_*sT2DM*_ and the cortical volume of the right superior occipital gyrus in all aMCI patients (cluster size: 108.05 mm^3^; cluster-level *P* < 0.05) (Figure 5A). The ROI-based correlation analysis is presented in Figure 5B.

### Mediation analysis

**FIGURE 6:**
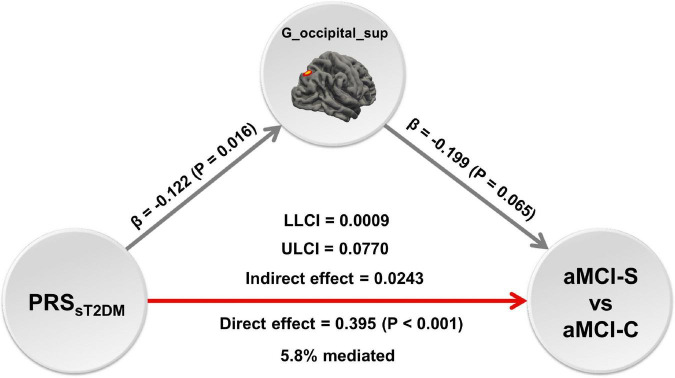
The mediation analysis. The mediation analysis shows that the cortical volume of right superior occipital gyrus mediates the predictive effect of the PRS_*sT2DM*_ on the conversion of amnestic mild cognitive impairment (aMCI), and the mediation effect could explain 5.8% of the variance. The red arrow shows positive effect, the gray arrows show negative effect. G_occipital_sup = the cortical volume of right superior occipital gyrus.

The mediation analysis showed that the cortical volume of the right superior occipital gyrus significantly mediated the association between the PRS_*sT2DM*_ and aMCI conversion (*P* < 0.05), and the mediation effect could explain 5.8% of the variance (Figure 6).

### Gene-based analyses

Using MAGMA, the 22,526 T2DM-specific genetic variants were fine-mapped into 6,238 genes based on genomic location (within a 5 kb window both upstream and downstream, 19,427 among the 22,526 SNPs were located inside genes). The genetic variants of the GWAS data of the Diabetes Genetics Replication and Meta-analysis (DIAGRM) were fine-mapped into 6,330 genes with a threshold of *P* < 0.05 using MAGMA. The overlapped 4,667 genes were used in the following analysis. Detailed fine-mapped genes are listed in Supplementary Table 3.

**FIGURE 7:**
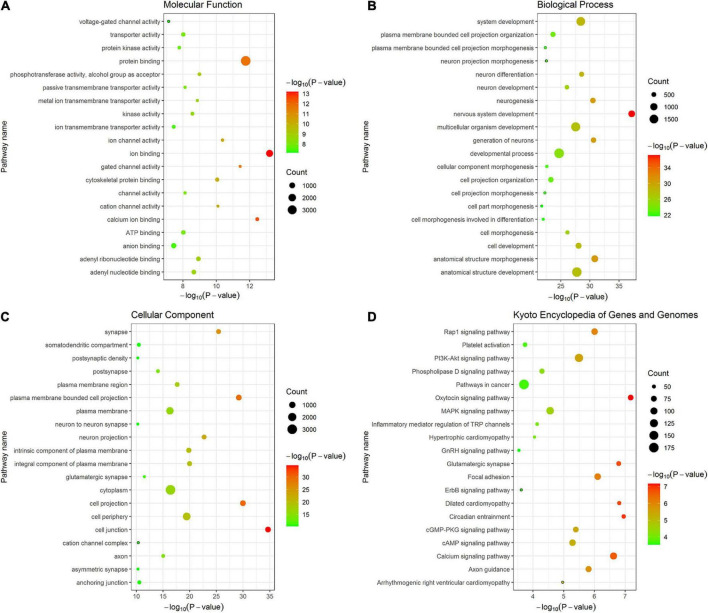
The top 20 items of GO and KEGG enrichment analyses of fine-mapped genes of T2DM-specific genetic variants. The size of each point represents fine-mapped gene numbers overlapped with GO or KEGG gene sets. The x-axis represents −log_10_ *P* (Bonferroni corrected). The y-axis represents GO or KEGG terms. **(A)** Molecular function **(B)** Biological processes **(C)** Cellular component **(D)** KEGG. GO, gene ontology; KEGG, kyoto encyclopedia of genes and genomes.

By performing GO and KEGG enrichment analyses, the 4,667 fine-mapped genes were significantly enriched in multiple GO items and pathways (*P* < 0.05, Bonferroni corrected) including molecular function GO items: ion binding (*P* = 6.49 × 10^–14^), calcium ion binding (*P* = 3.52 × 10^–13^), and protein binding (*P* = 1.72 × 10^–12^) (Figure 7A); biological processes GO items: nervous system development (*P* = 6.84 × 10^–38^), anatomical structure morphogenesis (*P* = 1.47 × 10^–31^), and generation of neurons (*P* = 2.45 × 10^–31^) (Figure 7B); cellular component GO items: cell junction (*P* = 2.11 × 10^–35^), cell projection (*P* = 1.08 × 10^–30^), and plasma membrane bounded cell projection (*P* = 6.30 × 10^–30^) (Figure 7C). In addition, the 6,267 genes were significantly enriched in oxytocin signaling pathway (*P* = 6.66 × 10^–8^), PI3K-Akt signaling pathway (*P* = 3.17 × 10^–6^), focal adhesion (*P* = 1.84 × 10^–7^), circadian entrainment (*P* = 1.12 × 10^–7^), and MAPK signaling pathway (*P* = 2.76 × 10^–5^) in KEGG (Figure 7D). Detailed enrichment analysis results are listed in Supplementary Table 4.

To assess cell-specific expression of the 4,667 fine-mapped genes, cell type-specific enrichment analysis was performed. Under different pSI thresholds (pSI = 0.05, 0.01, 0.001, and 0.0001, permutation corrected), which represents how likely a gene was specifically expressed in a given cell type relative to other cell types, the fine-mapped genes showed substantial overrepresentation, mainly in the cortical neuron (*P* = 0.011).

## Discussion

This study aimed to investigate whether T2DM-specific genetic risk can predict aMCI’s conversion to AD and further explore the underlying neurological mechanism. It was found that T2DM-specific polygenic genetic risk can predict aMCI’s conversion to AD, and the cortex volume of the right superior occipital gyrus might mediate this conversion. Furthermore, gene-based analyses suggested that T2DM-related genes were mainly enriched in the cortical neurons and might modulate ion and protein binding, neural development and generation, cell junction and projection, PI3K-Akt, and MAPK signaling pathway, which accelerated the aMCI’s conversion to AD by affecting Tau phosphorylation and Aβ accumulation (Karran et al., 2011).

### PRS_*sT2DM*_ independently predicts the conversion of amnestic mild cognitive impairment to Alzheimer’s disease

Alzheimer’s disease is a multifactorial neurodegenerative disorder that lacks a curative treatment. Therefore, early discovery, diagnosis, and treatment could significantly improve the patients’ prognosis. The aMCI is a type of syndrome between normal aging and AD (Winblad et al., 2004), patients with aMCI are at a high risk of converting to AD. AD risk genes such as APOEε4 can induce the deposition of tau protein and Aβ in and out of cells of brain regions, lead to reduced neuronal activity and loss of synapses, and accelerate brain atrophy in related areas (Kim et al., 2014), further promote the conversion of aMCI to AD. Our results confirmed Adams’ finding that the PRS_*AD*_ could predict the conversion of aMCI to AD (Adams et al., 2015).

It has been found that AD and T2DM patients share some pathological characteristics of the central nervous system. Hoyer (2000) found that brain metabolism changes, including impaired glucose utilization and energy metabolism, occurred in AD patients after the first clinical symptoms appeared. The metabolic impairment gradually worsens with AD’s progression (Hoyer et al., 1991). Therefore, some researchers have proposed that abnormal energy metabolism in AD patients may be caused by insulin resistance or weakened insulin action in the brain (Blass et al., 2002; Hoyer, 2004a,b). In addition, some studies have shown that T2DM risk genes could predict the conversion of aMCI to AD. For example, a longitudinal follow-up ADNI study showed that the SNP (rs391300) located on the serine racemic enzyme (SRR, a risk gene of T2DM) could predict the conversion of aMCI to AD (Girard et al., 2018). In this study, for the first time, we proved that PRS_*sT2DM*_ could predict the aMCI’s conversion after the shared iSNPs between PRS_*T2DM*_ and PRS_*AD*_ were removed, suggesting that the polygenic genetic risk of T2DM could be one of the risk factors for the conversion of aMCI to AD.

Type 2 diabetes mellitus risk genes can lead to impaired insulin signaling (Meur et al., 2010). The relationship between impaired brain insulin signaling and AD’s pathological changes may involve the following aspects: impaired insulin signaling transduction may lead to elevated glycogen synthase kinase-3β (GSK-3β) activity and oxidative stress, promoting tau protein’s hyperphosphorylation; impaired insulin signaling will inhibit the PI3-K-Akt signaling pathway, resulting in decreased neuronal activity and loss of synapses; insulin or IGF-1 can activate the Erk-MAPK pathway and promote the physiological processing and intracellular transport of β-APP to the plasma membrane (de la Monte and Wands, 2005). In addition, insulin receptors are usually highly expressed in brain areas related to cognition and memory, such as the cortex and hippocampus (Craft, 2009; Nisticò et al., 2012). Therefore, impaired insulin signaling-related tau protein increase and Aβ deposition in the brain may be the plausible neurobiological mechanism that the PRS_*T2DM*_ predicts the conversion of aMCI to AD.

### The relationship between PRS_*sT2DM*_ and brain structure

Our study found that the PRS_*sT2DM*_ was significantly negatively correlated with the GMV of the right superior frontal gyrus in the aMCI-C group. A meta-analysis showed that the GMV of the medial superior frontal gyrus of T2DM patients was significantly smaller than that of the controls (Liu et al., 2017). Furthermore, the GMV of the superior frontal gyrus in patients with T2DM combined with aMCI is significantly smaller than that of controls (Zhang et al., 2014). These results suggested that the superior frontal gyrus is prone to atrophy in T2DM patients. Our finding also indicated that the T2DM-related genetic risks might modulate the GMV of the superior frontal gyrus in T2DM patients.

In all aMCI patients, the PRS_*sT2DM*_ was significantly negatively correlated with the cortical volume of the right superior occipital gyrus. Many studies showed structural changes in the occipital lobe of T2DM patients, including gray matter atrophy (Zhang et al., 2014; Moulton et al., 2015) and decreased topological attributes (Qin et al., 2019), which demonstrated that the occipital lobe is a brain area prone to structural damages in T2DM patients. The occipital lobe can transmit visual information and cooperate with other brain regions to process and integrate visual and verbal information. It had been found that GMV of the occipital lobe in AD patients was significantly associated with attention function (Cromarty et al., 2018). Kunst et al. (2019) found that the atrophy of the occipital cortex can be used to distinguish between AD, aMCI, and healthy controls. Our study showed that the T2DM-related genetic risks might modulate the cortical volume of the right superior occipital gyrus in aMCI patients. Mediation analysis demonstrated that the cortical volume of the right superior occipital gyrus might mediate the predictive effect of the PRS_*sT2DM*_ on the conversion of aMCI to AD.

### Neurobiological mechanisms underlying the predictive effect of PRS_*sT2DM*_ on amnestic mild cognitive impairment conversion

To explore the neurobiological mechanism behind the predictive effect of T2DM-specific genetic variants on the aMCI conversion, we fine-mapped the T2DM-specific genetic variants to the genes and then performed the enrichment analyses.

Cell type enrichment analysis showed that these genes are mainly expressed in the cortical neuron. Gene GO enrichment analyses showed that these genes were primarily associated with ion and protein binding, neural development and generation, cell junction, and projection. These biological processes are known to be associated with AD. For example, metal ions binding to the Aβ peptide can promote aggregation of Aβ (Wallin et al., 2016, 2017). Insulin-like growth factor binding protein-2 (IGFBP-2) is associated with AD and brain atrophy (Lane et al., 2017). Neurogenesis represents an integral part of AD pathology (Mu and Gage, 2011). The projection of Hippocampal neurons may affect learning and memory (Milner and Veznedaroglu, 1993). The KEGG annotation revealed that fine-mapped genes were mainly related to the PI3K-Akt and MAPK signaling pathways. These enrichment analysis results suggested that these T2DM-related genetic variants are highly expressed in the cortical neurons, modulate ion and protein binding, neural development and generation, cell junction and projection, the PI3K-Akt and MAPK signaling pathway, and further affect Tau phosphorylation (Salkovic-Petrisic et al., 2006; Ke et al., 2009) and Aβ accumulation (Yang et al., 2014), accelerating the conversion of aMCI to AD.

Several limitations should be mentioned in this study. First, the PRS was calculated with an additive effect by default; we did not consider the influence of other genetic effects on the results. Second, we did not control some clinical situations, such as vasculopathy, during the predicting analysis because of insufficient data. Further study should be performed to clarify the impact of clinical status on the results. Third, the cell type-specific expression analysis was based on the cortex of the mouse because no public cell type-specific expression data in the human cortex can be obtained.

## Conclusion

This study aims to evaluate whether T2DM-specific genetic variants can predict the conversion of aMCI to AD and further explore the underlying neurological mechanism. The results showed that the PRS_*sT2DM*_ could independently predict aMCI conversion to AD. The cortical volume of the right superior occipital gyrus may mediate the predictive effect of the PRS_*sT2DM*_ on the conversion of aMCI to AD. T2DM-related genetic genes are highly expressed in the cortical neurons, may modulate ion and protein binding, neural development and generation, cell junction and projection, and the PI3K-Akt and MAPK signaling pathways, which is related to Tau phosphorylation and Aβ accumulation, accelerating the conversion of aMCI to AD.

## Data availability statement

The datasets presented in this study can be found in online repositories. The names of the repository/repositories and accession number(s) can be found below: http://adni.loni.usc.edu/.

## Author contributions

JY, ZW, JX, WQ, and QZ designed the research. JY, ZW, and QZ wrote the manuscript. QZ was the guarantor of this work and took responsibility for the integrity of the data and the accuracy of the data analysis. All authors analyzed the data and approved the submitted version.

## Footnotes

1
http://adni.loni.ucla.edu

2
http://www.fil.ion.ucl.ac.uk/spm

3
http://dbm.neuro.uni-jena.de/cat12/

4
http://surfer.nmr.mgh.harvard.edu/

5
http://freesurfer.net/fswiki/Edits

6
https://genome.ucsc.edu/

7
https://biit.cs.ut.ee/gprofiler/gost

8
http://genetics.wustl.edu/jdlab/csea-tool-2/
