# Supplementary material for: Prediction value of the genetic risk of type 2 diabetes on the amnestic mild cognitive impairment conversion to Alzheimer’s disease
Source: Front Aging Neurosci. 2022 Sep 15;14:964463. doi: 10.3389/fnagi.2022.964463 (PMC9521369; doi:10.3389/fnagi.2022.964463)
Supplement: Supplementary file 1 [file Table_1.docx]

| **Supplemental Table 1. Intergroup comparisons of** **Aβ and TAU level in the CSF** | | | | |
| --- | --- | --- | --- | --- |
| **Variables** | **aMCI-S**  **(n = 185)** | **aMCI-C**  **(n = 114)** | **Z** | **P** |
| **Aβ** | 1102.85 | 743.74 | -7.28 | **<0.001** |
| **TAU** | 256.95 | 338.50 | -6.16 | **<0.001** |
| Mann-Whitney U test was used to compare differences between two groups. | | | | |
